# Recent Advances in the Treatment of Consumption

**Published:** 1888-02

**Authors:** 


### RECENT ADVANCES IN THE TREATMENT OF CONSUMPTION.

No disease on the list is so thoroughly the *bete noir* of medicine as is consumption. If exhaustive study, careful experiment, close observation and intense desire have been especially devoted to one ailment, that ailment is consumption, and yet it stalks through the land quite as deadly in its visitations, to all appearances, as it ever has in its history. The prospect of discovering some agent, or devising some means of staying its ravages is not encouraging. In spite of all that has been done, the physician who is consulted by a consumptive goes about his duty of prescribing medicinally and hygienically, in a sort of perfunctionary manner, having little hope that his most devoted efforts can do aught towards staying the course of disease. No practitioner would, however, feel himself justified in pursuing the purely expectant plan of treatment, and it becomes him to be familiar with the most recent "advances" in the therapeutics of the affection. These are admirably set forth in a recent article by Dr. S. S. Cohen, of Philadelphia, in the *Medical News*. In the article the author declares that the objective therapeutic point may be summed up in one word—Nutrition. In his efforts to accomplish this he gives prom nence to Debove's plan of forced feeding, or the introduction into the stomach of a flexible tube connected with a funnel through which is passed the aliment to be administered. The taste of the food thus being a matter of no importance, feeding can be conducted to the limits of the digestive capacity of the stomach. Debove's meat powder has usually been given, but milk, eggs, soups and farinaceous powders may be employed instead or in addition. A mixture which has found favor with the author consists of a quart of eggs, fifteen grains of scale pepsin and thirty drops of dilute hydrochloric acid. This mixture may be introduced twice a day Fried foods of all kinds, pastry and other such indigestible matter must be discarded.

The problem of nourishing the consumptive divides itself naturally into three parts: First, the preparation of the digestive tract, to elaborate and to absorb the chylous fluids—primary assimilation. Second, the promotion of the complex process of the breaking down and displacement of imperfect tissues and effete products, and replacement by new and vigorous tissues, with evolution of forces required in the economy; *i. e.*, metabolism—secondary assimilation; and third, the promotion of the excretion of waste products.

The first disideratum is endeavored to be secured by methods which cleanse, disinfect, and stimulate the digestive canal; varied in detail according to circumstances. When we have reason to suppose, for example, that a sluggish gastric catarrh interferes with digestion, washing out the stomach may be practiced with good effect. The procedure is quite simple. A stomach tube of similar material to French catheter tubing, about 28 inches long, and from ¼ inch to 7-16 of an inch in diameter, is attached by a short section of glass tubing, to a soft rubber tube about a yard long, in the extremity of which is inserted a hard rubber funnel of about six-ounce capacity The stomach-tube having been dipped into water or warm milk, is introduced into the œsophagus and propelled by succesive pushes, or swallowed by the patient ; and the fnnnel being sufficiently elevated, from a pint to a quart or more of warm water (100° F.), in which is dissolved a drachm or two of borax, table salt, or baking soda, is slowly poured into the funnel. As the last of the fluid is passed out of the funnel, the latter is rapidly inverted over a receptacle on the floor, and the contents of the stomach are thus siphoned out. The manœuvre is repeated until the return water is clear. This process, called lavage, which, as already stated, suggested gavage, and is practiced in much the same manner, leaves the gastric mucous membrane in excellent condition for digestion and absorption. It may be immediately followed by gavage, as recommended by Dujardin-Beaumetz. The drinking of half a pint to a pint of hot water, half an hour to an hour before meal time, will sometimes accomplish much the same purpose, and is, of course, less troublesome.

When a condition of septic fermentation is believed to interfere with digestion, a suitable antiseptic agent, such as carbon-disulphide water or solution of hydrogen diohide, may be introduced into the lavage solution, and a portion allowed to remain a few minutes in the stomach ; or creosote, carbolic acid, iodoform, the solutions mentioned, or other agents may be administerd in the ordinary way. When the intestinal canal is believed to be the seat of trouble, we may attempt to wash it, indirectly by potations of hot water, or to medicate it with creosote, bismuth, sulphocarbolates, mercurials, iodoform, sulphides, naphthalin, or other appropriate drugs. Dr. Cohen has reason to believe from the effect produced upon some cases of phthisis attended with diarahœa, that the injection per rectum, of hydrogen sulphide, directly or indirectly arrests fermentations in the small intestine.

To aid digestion, stimulate digestive secretion, and promote absorption, in addition to the measures already referred to, preparations of malt, Hoffman's anodyne, bitter tonics, nux vomica, arsenic, preferably Fowler's solution, iron, nitro-hydrochloric, nitric, and phosphoric acids, trinitrin, and other appropriate medication may be employed when indicated.

Nutriment being administered, digested, and absorbed into the blood, must be converted into vital forces, and into tissue. Exercise and respiration are the natural means to effect this. "Respiration," said Arbuthnot, "is the second digestion."

When the patient is able to carry out the instructions, and when there is a sufficiency of unimpaired lung tissue, respiratory gymnastics, and voluntary forced respiration may suffice. Ordinarily, however, these measures will not be efficient, and must be replaced or supplemented by a method which affords mechanical assistance to respiration, independent of voluntary exertion. This method offers itself in the inhalation of compressed air, a subject which will always be associated with the name of its great promoter, Waldenburg. The air is inspired under an excess pressure, gradually increased from 1-80—1-60 up to 1-40 or 1-20 of an atmosphere; sometimes into rarefied air. The inhalations are administered once or twice daily. At each period, ten or fifteen, twenty-five or hirty, up to one hundred or more respiratory acts are completed in five to fifteen minutes, and the process is repeated after an interval of about ten minutes.

Our general tonic medication, exercise, orced respiration, etc., will of course, assist directly, and indirectly, as emunctorial stimulants. The daily sponge bath, which, to the well, is a matter of comfort and cleanliness, becomes to the consumptive a measure of therapeusis. The drinking of water, preferably hot, is again applicable as the best of diuretics and a potent diaphoretic. Lemon juice and sugar may be added to render it more palatable, the former indeed, increasing its value as a diuretic. Nitro-hydrochloric acid is among the best hepatic stimulants in this connection. An enema is ordinarily the best method of emptying the bowels. To overcome intestinal torpor the same measures employed under other circumstances, nux vomica, belladonna, faradism, etc, may be resorted to. Among the preferable cholagogue cathartics, are podophylliin and rhubarb.

The indications thus far considered may be fulfilled in the generality of cases by the following routine :

1. An abundant and proper diet, as already discussed ; gavage if necessary.

2. The drinking of hot water, or hot lemonade ; lavage, if necessary.

3. Moderate open-air exercise : respiratory gymnastics ; daily inhalations of compressed air.

4. The administration of some such pill as this, three or four times a day: Iodoform, 1 to 2 grains, creosote one-half minim to one minim ; to which may sometimes be added reduced iron, 1 grain, or arsenious acid, 1-60 to 1-20th grain, the pill being made up with glucose, crude petroleum, or extract of licorice, with the addition, if indicated, of some bitter extract, such as gentian, cinchona, or nux vomica, and dispensed in capsule. Among other useful prescriptions may be cited, when iron is indicated: Compound syrup of phosphate of iron (Parrish); tincture of chloride of iron, dilute phosphoric acid, and Churchill's syrup of hypophosphites and iron, etc. Iron seems to be better borne by the stomach, and to be more readily appropriated by the red blood globules when inhalation of compressed air is practiced. Cardiac weakness, excessive febrile action, and other conditions may call for appropriate medication.

The Bergeon treatment by rectal injections of sulphuretted hydrogen gas, the author holds to be still on trial. He has no hope for it as a bacteriacide, but as a remedy for those local conditions which permit the microbe to find a suitable habitat in the lungs.—*Medical Age.*

**Iron.**—It will precipitate the gastric juice taken before meals, therefore take it when there is something in the stomach to prevent this. It is not known how it gets into the circulation, because it is not seen to go out. In any case, give it *with meals.*

